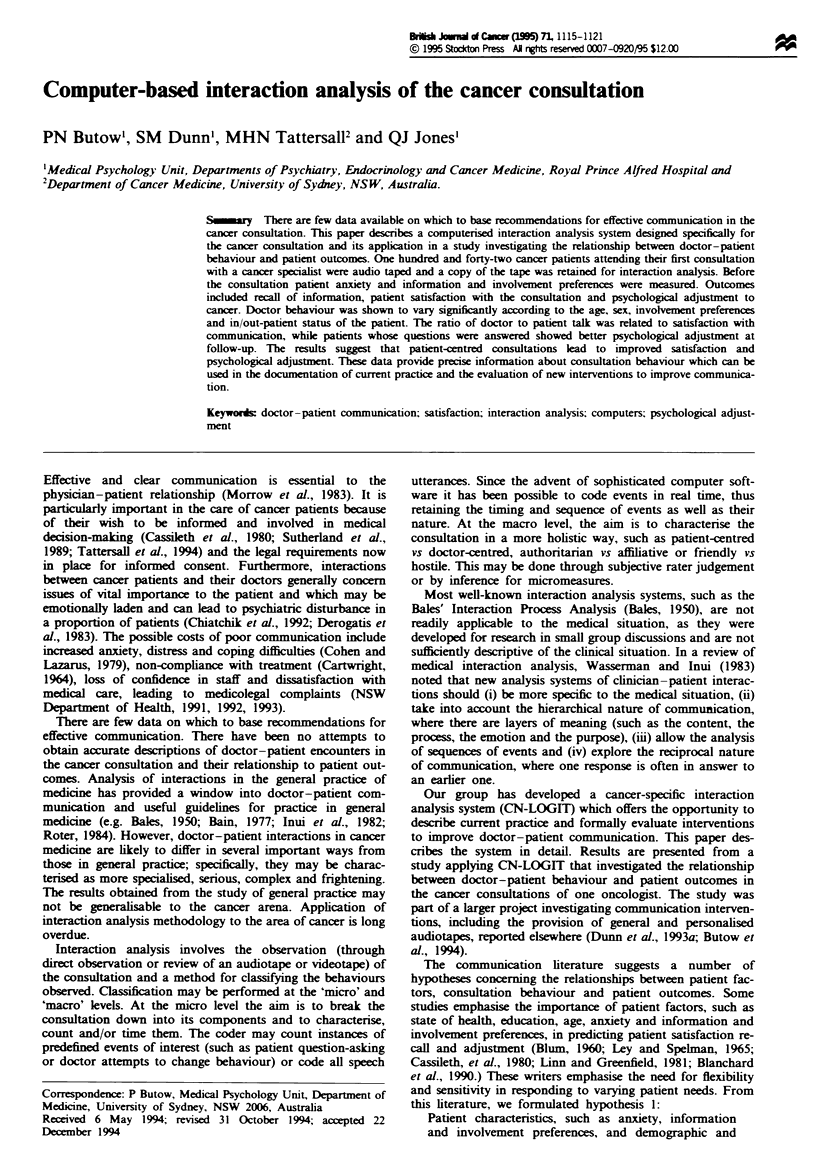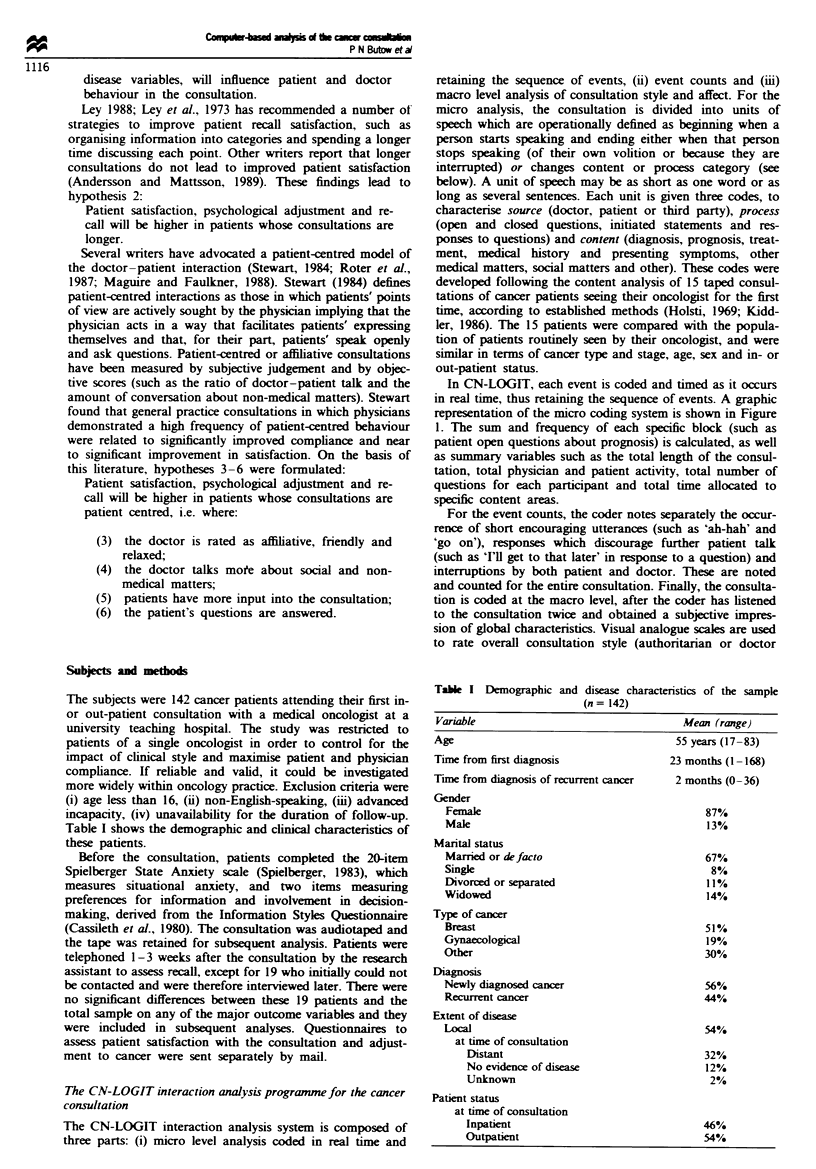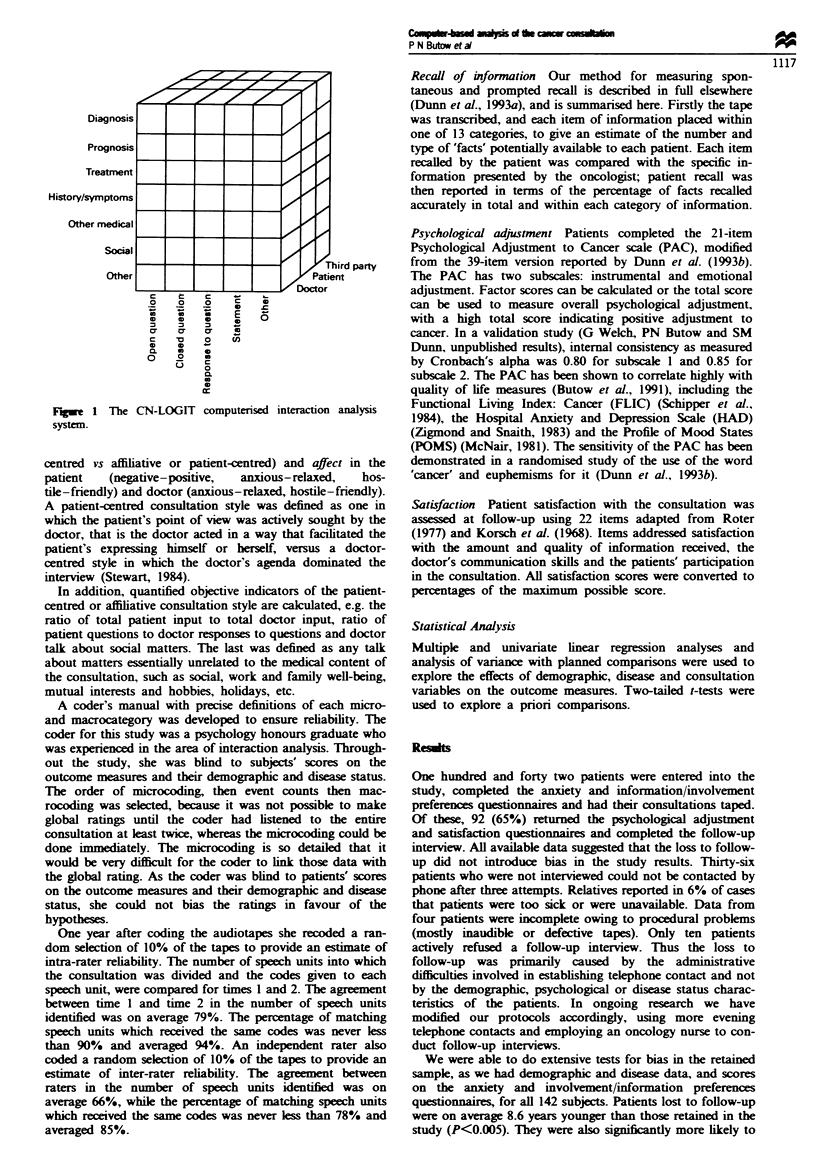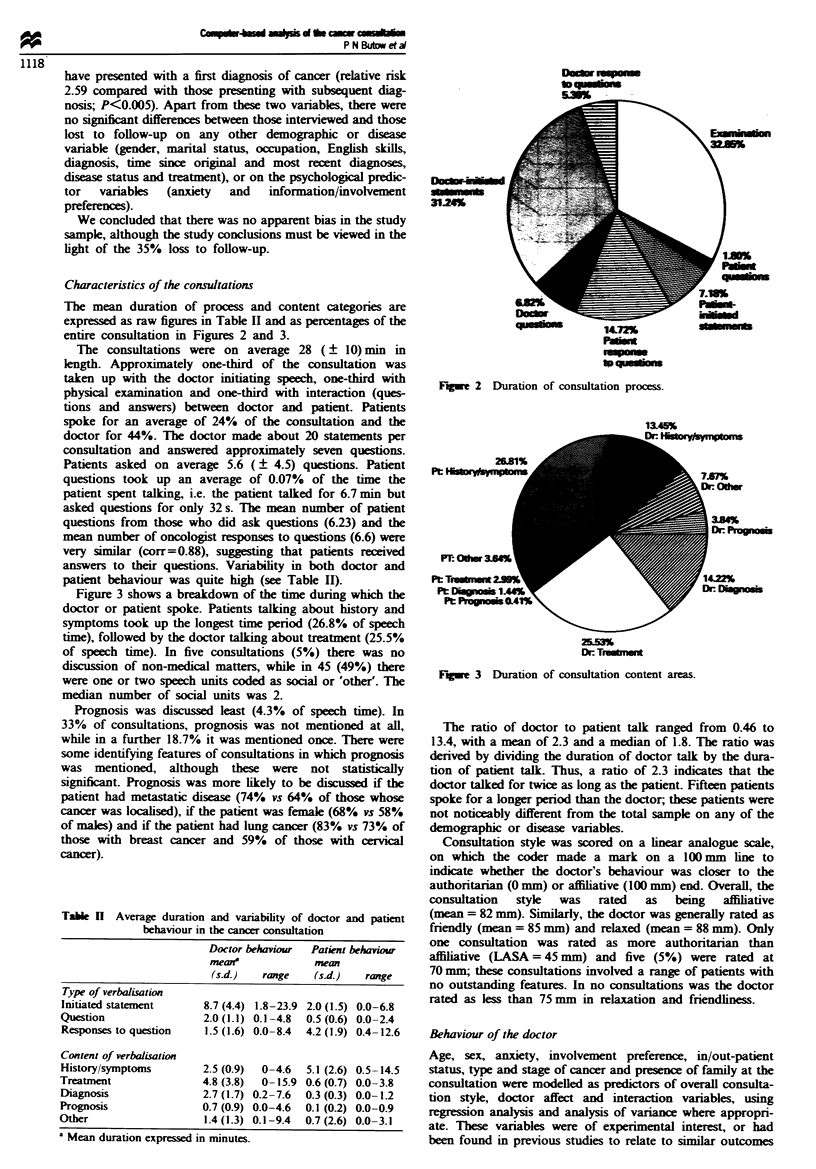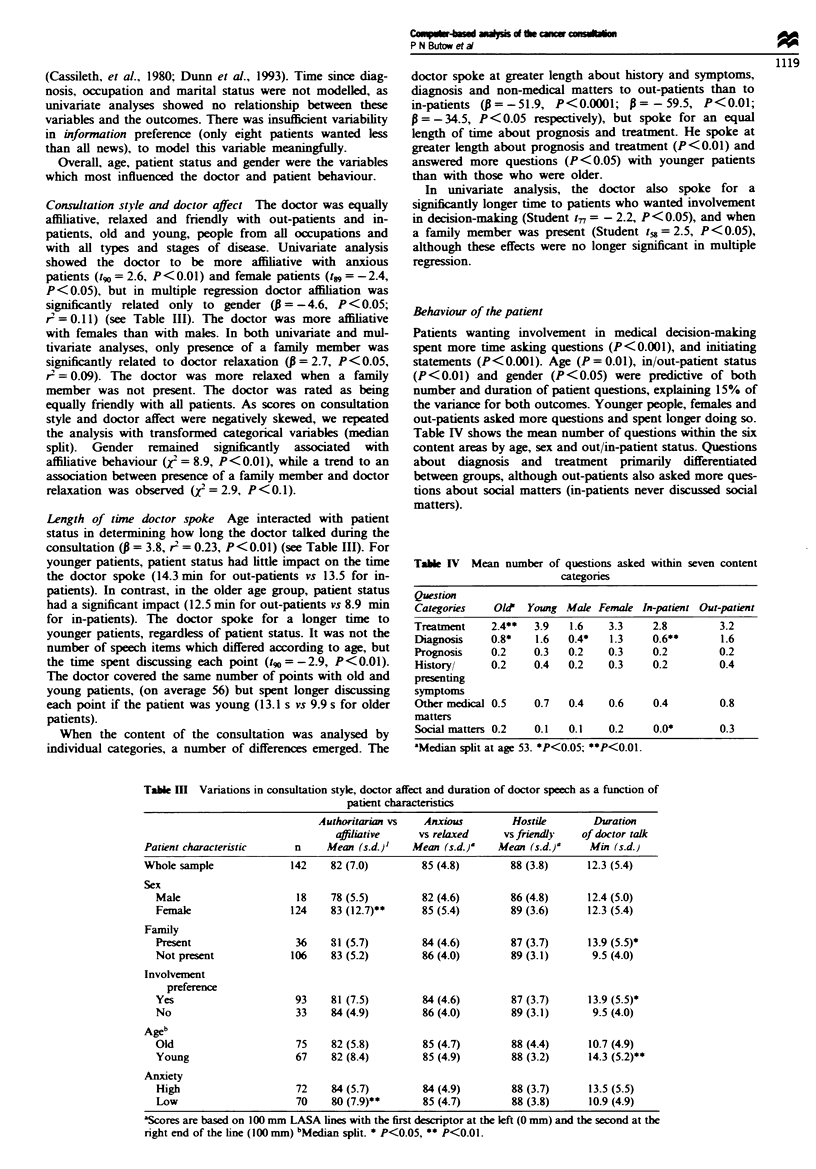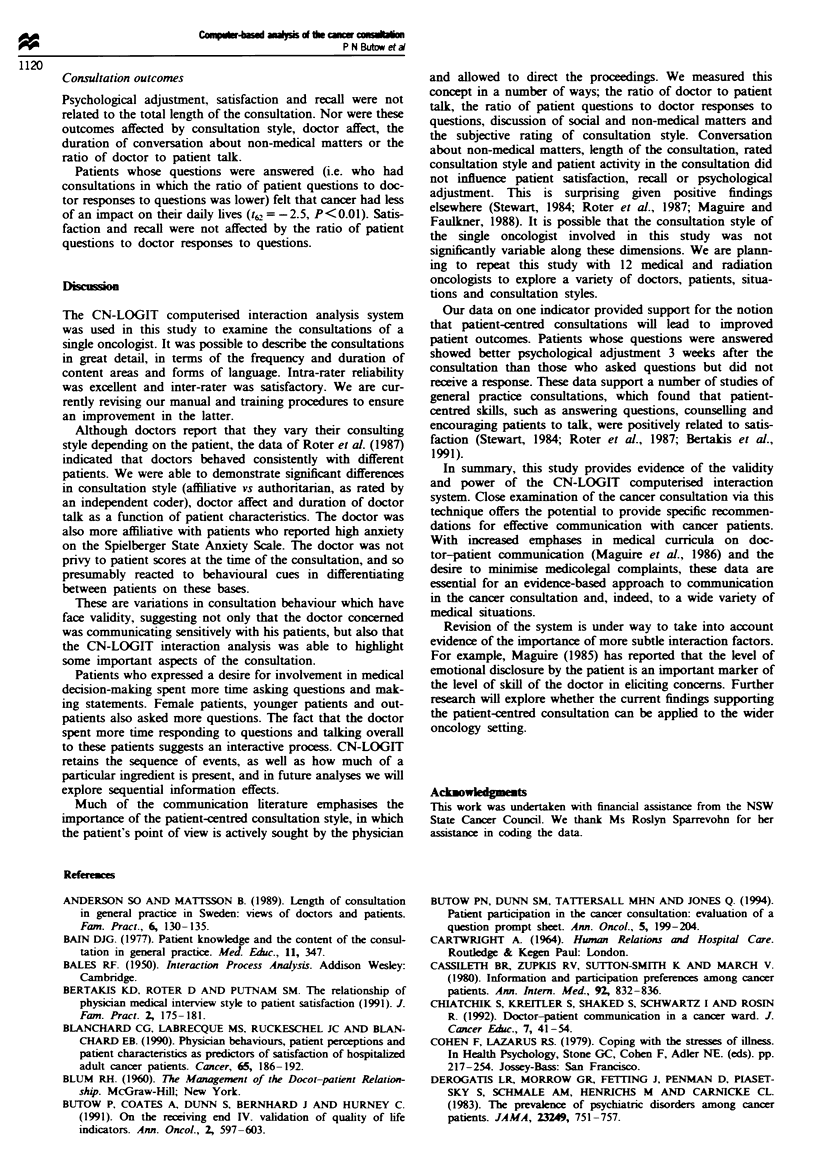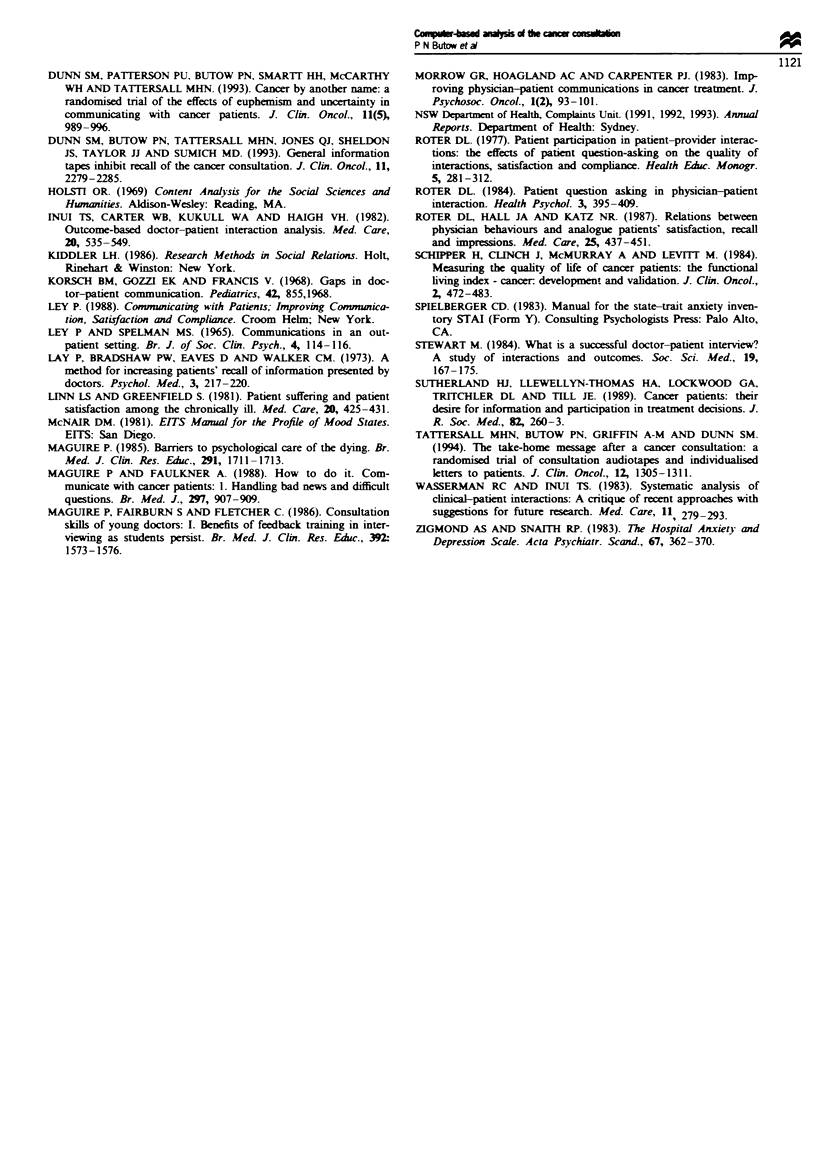# Computer-based interaction analysis of the cancer consultation.

**DOI:** 10.1038/bjc.1995.216

**Published:** 1995-05

**Authors:** P. N. Butow, S. M. Dunn, M. H. Tattersall, Q. J. Jones

**Affiliations:** Department of Psychiatry, Royal Prince Alfred Hospital, NSW, Australia.

## Abstract

There are few data available on which to base recommendations for effective communication in the cancer consultation. This paper describes a computerised interaction analysis system designed specifically for the cancer consultation and its application in a study investigating the relationship between doctor-patient behaviour and patient outcomes. One hundred and forty-two cancer patients attending their first consultation with a cancer specialist were audio taped and a copy of the tape was retained for interaction analysis. Before the consultation patient anxiety and information and involvement preferences were measured. Outcomes included recall of information, patient satisfaction with the consultation and psychological adjustment to cancer. Doctor behaviour was shown to vary significantly according to the age, sex, involvement preferences and in/out-patient status of the patient. The ratio of doctor to patient talk was related to satisfaction with communication, while patients whose questions were answered showed better psychological adjustment at follow-up. The results suggest that patient-centred consultations lead to improved satisfaction and psychological adjustment. These data provide precise information about consultation behaviour which can be used in the documentation of current practice and the evaluation of new interventions to improve communication.


British Journal of Cancer (1995) 71, 1115-1121

© 1995 Stockton Press All rights reserved 0007-0920/95 $12.00

### Computer-based interaction analysis of the cancer consultation

PN Butow[1], SM Dunn[1], MHN Tattersall[2] and QJ Jones[1]

[1]*Medical Psychology Unit, Departments of Psychiatry, Endocrinology and Cancer Medicine, Royal Prince Alfred Hospital and* [2]*Department of Cancer Medicine, University of Sydney, NSW, Australia.*

**Summary** There are few data available on which to base recommendations for effective communication in the cancer consultation. This paper describes a computerised interaction analysis system designed specifically for the cancer consultation and its application in a study investigating the relationship between doctor–patient behaviour and patient outcomes. One hundred and forty-two cancer patients attending their first consultation with a cancer specialist were audio taped and a copy of the tape was retained for interaction analysis. Before the consultation patient anxiety and information and involvement preferences were measured. Outcomes included recall of information, patient satisfaction with the consultation and psychological adjustment to cancer. Doctor behaviour was shown to vary significantly according to the age, sex, involvement preferences and in/out-patient status of the patient. The ratio of doctor to patient talk was related to satisfaction with communication, while patients whose questions were answered showed better psychological adjustment at follow-up. The results suggest that patient-centred consultations lead to improved satisfaction and psychological adjustment. These data provide precise information about consultation behaviour which can be used in the documentation of current practice and the evaluation of new interventions to improve communication.

**Keywords:** doctor–patient communication; satisfaction; interaction analysis; computers; psychological adjustment

Effective and clear communication is essential to the physician–patient relationship (Morrow *et al.*, 1983). It is particularly important in the care of cancer patients because of their wish to be informed and involved in medical decision-making (Cassileth *et al.*, 1980; Sutherland *et al.*, 1989; Tattersall *et al.*, 1994) and the legal requirements now in place for informed consent. Furthermore, interactions between cancer patients and their doctors generally concern issues of vital importance to the patient and which may be emotionally laden and can lead to psychiatric disturbance in a proportion of patients (Chiatchik *et al.*, 1992; Derogatis *et al.*, 1983). The possible costs of poor communication include increased anxiety, distress and coping difficulties (Cohen and Lazarus, 1979), non-compliance with treatment (Cartwright, 1964), loss of confidence in staff and dissatisfaction with medical care, leading to medicolegal complaints (NSW Department of Health, 1991, 1992, 1993).

There are few data on which to base recommendations for effective communication. There have been no attempts to obtain accurate descriptions of doctor–patient encounters in the cancer consultation and their relationship to patient outcomes. Analysis of interactions in the general practice of medicine has provided a window into doctor–patient communication and useful guidelines for practice in general medicine (e.g. Bales, 1950; Bain, 1977; Inui *et al.*, 1982; Roter, 1984). However, doctor–patient interactions in cancer medicine are likely to differ in several important ways from those in general practice; specifically, they may be characterised as more specialised, serious, complex and frightening. The results obtained from the study of general practice may not be generalisable to the cancer arena. Application of interaction analysis methodology to the area of cancer is long overdue.

Interaction analysis involves the observation (through direct observation or review of an audiotape or videotape) of the consultation and a method for classifying the behaviours observed. Classification may be performed at the 'micro' and 'macro' levels. At the micro level the aim is to break the consultation down into its components and to characterise, count and/or time them. The coder may count instances of predefined events of interest (such as patient question-asking or doctor attempts to change behaviour) or code all speech utterances. Since the advent of sophisticated computer software it has been possible to code events in real time, thus retaining the timing and sequence of events as well as their nature. At the macro level, the aim is to characterise the consultation in a more holistic way, such as patient-centred *vs* doctor-centred, authoritarian *vs* affiliative or friendly *vs* hostile. This may be done through subjective rater judgement or by inference for micromeasures.

Most well-known interaction analysis systems, such as the Bales' Interaction Process Analysis (Bales, 1950), are not readily applicable to the medical situation, as they were developed for research in small group discussions and are not sufficiently descriptive of the clinical situation. In a review of medical interaction analysis, Wasserman and Inui (1983) noted that new analysis systems of clinician–patient interactions should (i) be more specific to the medical situation, (ii) take into account the hierarchical nature of communication, where there are layers of meaning (such as the content, the process, the emotion and the purpose), (iii) allow the analysis of sequences of events and (iv) explore the reciprocal nature of communication, where one response is often in answer to an earlier one.

Our group has developed a cancer-specific interaction analysis system (CN-LOGIT) which offers the opportunity to describe current practice and formally evaluate interventions to improve doctor–patient communication. This paper describes the system in detail. Results are presented from a study applying CN-LOGIT that investigated the relationship between doctor–patient behaviour and patient outcomes in the cancer consultations of one oncologist. The study was part of a larger project investigating communication interventions, including the provision of general and personalised audiotapes, reported elsewhere (Dunn *et al.*, 1993*a*; Butow *et al.*, 1994).

The communication literature suggests a number of hypotheses concerning the relationships between patient factors, consultation behaviour and patient outcomes. Some studies emphasise the importance of patient factors, such as state of health, education, age, anxiety and information and involvement preferences, in predicting patient satisfaction recall and adjustment (Blum, 1960; Ley and Spelman, 1965; Cassileth, *et al.*, 1980; Linn and Greenfield, 1981; Blanchard *et al.*, 1990.) These writers emphasise the need for flexibility and sensitivity in responding to varying patient needs. From this literature, we formulated hypothesis 1:

Patient characteristics, such as anxiety, information and involvement preferences, and demographic and

Correspondence: P Butow, Medical Psychology Unit, Department of Medicine, University of Sydney, NSW 2006, Australia
Received 6 May 1994; revised 31 October 1994; accepted 22 December 1994

disease variables, will influence patient and doctor behaviour in the consultation.

Ley 1988; Ley *et al*., 1973 has recommended a number of strategies to improve patient recall satisfaction, such as organising information into categories and spending a longer time discussing each point. Other writers report that longer consultations do not lead to improved patient satisfaction (Andersson and Mattsson, 1989). These findings lead to hypothesis 2:

Patient satisfaction, psychological adjustment and recall will be higher in patients whose consultations are longer.

Several writers have advocated a patient-centred model of the doctor–patient interaction (Stewart, 1984; Roter *et al*., 1987; Maguire and Faulkner, 1988). Stewart (1984) defines patient-centred interactions as those in which patients' points of view are actively sought by the physician implying that the physician acts in a way that facilitates patients' expressing themselves and that, for their part, patients' speak openly and ask questions. Patient-centred or affiliative consultations have been measured by subjective judgement and by objective scores (such as the ratio of doctor–patient talk and the amount of conversation about non-medical matters). Stewart found that general practice consultations in which physicians demonstrated a high frequency of patient-centred behaviour were related to significantly improved compliance and near to significant improvement in satisfaction. On the basis of this literature, hypotheses 3–6 were formulated:

Patient satisfaction, psychological adjustment and recall will be higher in patients whose consultations are patient centred, i.e. where:

(3) the doctor is rated as affiliative, friendly and relaxed;
(4) the doctor talks more about social and non-medical matters;
(5) patients have more input into the consultation;
(6) the patient's questions are answered.

### Subjects and methods

The subjects were 142 cancer patients attending their first in- or out-patient consultation with a medical oncologist at a university teaching hospital. The study was restricted to patients of a single oncologist in order to control for the impact of clinical style and maximise patient and physician compliance. If reliable and valid, it could be investigated more widely within oncology practice. Exclusion criteria were (i) age less than 16, (ii) non-English-speaking, (iii) advanced incapacity, (iv) unavailability for the duration of follow-up. Table I shows the demographic and clinical characteristics of these patients.

Before the consultation, patients completed the 20-item Spielberger State Anxiety scale (Spielberger, 1983), which measures situational anxiety, and two items measuring preferences for information and involvement in decision-making, derived from the Information Styles Questionnaire (Cassileth *et al*., 1980). The consultation was audiotaped and the tape was retained for subsequent analysis. Patients were telephoned 1–3 weeks after the consultation by the research assistant to assess recall, except for 19 who initially could not be contacted and were therefore interviewed later. There were no significant differences between these 19 patients and the total sample on any of the major outcome variables and they were included in subsequent analyses. Questionnaires to assess patient satisfaction with the consultation and adjustment to cancer were sent separately by mail.

### *The CN-LOGIT interaction analysis programme for the cancer consultation*

The CN-LOGIT interaction analysis system is composed of three parts: (i) micro level analysis coded in real time and retaining the sequence of events, (ii) event counts and (iii) macro level analysis of consultation style and affect. For the micro analysis, the consultation is divided into units of speech which are operationally defined as beginning when a person starts speaking and ending either when that person stops speaking (of their own volition or because they are interrupted) *or* changes content or process category (see below). A unit of speech may be as short as one word or as long as several sentences. Each unit is given three codes, to characterise *source* (doctor, patient or third party), *process* (open and closed questions, initiated statements and responses to questions) and *content* (diagnosis, prognosis, treatment, medical history and presenting symptoms, other medical matters, social matters and other). These codes were developed following the content analysis of 15 taped consultations of cancer patients seeing their oncologist for the first time, according to established methods (Holsti, 1969; Kiddler, 1986). The 15 patients were compared with the population of patients routinely seen by their oncologist, and were similar in terms of cancer type and stage, age, sex and in- or out-patient status.

In CN-LOGIT, each event is coded and timed as it occurs in real time, thus retaining the sequence of events. A graphic representation of the micro coding system is shown in Figure 1. The sum and frequency of each specific block (such as patient open questions about prognosis) is calculated, as well as summary variables such as the total length of the consultation, total physician and patient activity, total number of questions for each participant and total time allocated to specific content areas.

For the event counts, the coder notes separately the occurrence of short encouraging utterances (such as 'ah-hah' and 'go on'), responses which discourage further patient talk (such as 'I'll get to that later' in response to a question) and interruptions by both patient and doctor. These are noted and counted for the entire consultation. Finally, the consultation is coded at the macro level, after the coder has listened to the consultation twice and obtained a subjective impression of global characteristics. Visual analogue scales are used to rate overall consultation style (authoritarian or doctor

**Table I** Demographic and disease characteristics of the sample ($n = 142$)

| *Variable* | *Mean (range)* |
|---|---|
| Age | 55 years (17–83) |
| Time from first diagnosis | 23 months (1–168) |
| Time from diagnosis of recurrent cancer | 2 months (0–36) |
| Gender | |
| Female | 87% |
| Male | 13% |
| Marital status | |
| Married or *de facto* | 67% |
| Single | 8% |
| Divorced or separated | 11% |
| Widowed | 14% |
| Type of cancer | |
| Breast | 51% |
| Gynaecological | 19% |
| Other | 30% |
| Diagnosis | |
| Newly diagnosed cancer | 56% |
| Recurrent cancer | 44% |
| Extent of disease | |
| Local | 54% |
| at time of consultation | |
| Distant | 32% |
| No evidence of disease | 12% |
| Unknown | 2% |
| Patient status | |
| at time of consultation | |
| Inpatient | 46% |
| Outpatient | 54% |

**Figure 1** The CN-LOGIT computerised interaction analysis system.

centred *vs* affiliative or patient-centred) and *affect* in the patient (negative–positive, anxious–relaxed, hostile–friendly) and doctor (anxious–relaxed, hostile–friendly). A patient-centred consultation style was defined as one in which the patient's point of view was actively sought by the doctor, that is the doctor acted in a way that facilitated the patient's expressing himself or herself, versus a doctor-centred style in which the doctor's agenda dominated the interview (Stewart, 1984).

In addition, quantified objective indicators of the patient-centred or affiliative consultation style are calculated, e.g. the ratio of total patient input to total doctor input, ratio of patient questions to doctor responses to questions and doctor talk about social matters. The last was defined as any talk about matters essentially unrelated to the medical content of the consultation, such as social, work and family well-being, mutual interests and hobbies, holidays, etc.

A coder's manual with precise definitions of each micro- and macrocategory was developed to ensure reliability. The coder for this study was a psychology honours graduate who was experienced in the area of interaction analysis. Throughout the study, she was blind to subjects' scores on the outcome measures and their demographic and disease status. The order of microcoding, then event counts then macrocoding was selected, because it was not possible to make global ratings until the coder had listened to the entire consultation at least twice, whereas the microcoding could be done immediately. The microcoding is so detailed that it would be very difficult for the coder to link those data with the global rating. As the coder was blind to patients' scores on the outcome measures and their demographic and disease status, she could not bias the ratings in favour of the hypotheses.

One year after coding the audiotapes she recoded a random selection of 10% of the tapes to provide an estimate of intra-rater reliability. The number of speech units into which the consultation was divided and the codes given to each speech unit, were compared for times 1 and 2. The agreement between time 1 and time 2 in the number of speech units identified was on average 79%. The percentage of matching speech units which received the same codes was never less than 90% and averaged 94%. An independent rater also coded a random selection of 10% of the tapes to provide an estimate of inter-rater reliability. The agreement between raters in the number of speech units identified was on average 66%, while the percentage of matching speech units which received the same codes was never less than 78% and averaged 85%.

*Recall of information* Our method for measuring spontaneous and prompted recall is described in full elsewhere (Dunn *et al*., 1993*a*), and is summarised here. Firstly the tape was transcribed, and each item of information placed within one of 13 categories, to give an estimate of the number and type of 'facts' potentially available to each patient. Each item recalled by the patient was compared with the specific information presented by the oncologist; patient recall was then reported in terms of the percentage of facts recalled accurately in total and within each category of information.

*Psychological adjustment* Patients completed the 21-item Psychological Adjustment to Cancer scale (PAC), modified from the 39-item version reported by Dunn *et al.* (1993*b*). The PAC has two subscales: instrumental and emotional adjustment. Factor scores can be calculated or the total score can be used to measure overall psychological adjustment, with a high total score indicating positive adjustment to cancer. In a validation study (G Welch, PN Butow and SM Dunn, unpublished results), internal consistency as measured by Cronbach's alpha was 0.80 for subscale 1 and 0.85 for subscale 2. The PAC has been shown to correlate highly with quality of life measures (Butow *et al*., 1991), including the Functional Living Index: Cancer (FLIC) (Schipper *et al*., 1984), the Hospital Anxiety and Depression Scale (HAD) (Zigmond and Snaith, 1983) and the Profile of Mood States (POMS) (McNair, 1981). The sensitivity of the PAC has been demonstrated in a randomised study of the use of the word 'cancer' and euphemisms for it (Dunn *et al*., 1993*b*).

*Satisfaction* Patient satisfaction with the consultation was assessed at follow-up using 22 items adapted from Roter (1977) and Korsch *et al.* (1968). Items addressed satisfaction with the amount and quality of information received, the doctor's communication skills and the patients' participation in the consultation. All satisfaction scores were converted to percentages of the maximum possible score.

### *Statistical Analysis*

Multiple and univariate linear regression analyses and analysis of variance with planned comparisons were used to explore the effects of demographic, disease and consultation variables on the outcome measures. Two-tailed *t*-tests were used to explore a priori comparisons.

### Results

One hundred and forty two patients were entered into the study, completed the anxiety and information/involvement preferences questionnaires and had their consultations taped. Of these, 92 (65%) returned the psychological adjustment and satisfaction questionnaires and completed the follow-up interview. All available data suggested that the loss to follow-up did not introduce bias in the study results. Thirty-six patients who were not interviewed could not be contacted by phone after three attempts. Relatives reported in 6% of cases that patients were too sick or were unavailable. Data from four patients were incomplete owing to procedural problems (mostly inaudible or defective tapes). Only ten patients actively refused a follow-up interview. Thus the loss to follow-up was primarily caused by the administrative difficulties involved in establishing telephone contact and not by the demographic, psychological or disease status characteristics of the patients. In ongoing research we have modified our protocols accordingly, using more evening telephone contacts and employing an oncology nurse to conduct follow-up interviews.

We were able to do extensive tests for bias in the retained sample, as we had demographic and disease data, and scores on the anxiety and involvement/information preferences questionnaires, for all 142 subjects. Patients lost to follow-up were on average 8.6 years younger than those retained in the study ($P<0.005$). They were also significantly more likely to have presented with a first diagnosis of cancer (relative risk 2.59 compared with those presenting with subsequent diagnosis; $P<0.005$). Apart from these two variables, there were no significant differences between those interviewed and those lost to follow-up on any other demographic or disease variable (gender, marital status, occupation, English skills, diagnosis, time since original and most recent diagnoses, disease status and treatment), or on the psychological predictor variables (anxiety and information/involvement preferences).

We concluded that there was no apparent bias in the study sample, although the study conclusions must be viewed in the light of the 35% loss to follow-up.

### *Characteristics of the consultations*

The mean duration of process and content categories are expressed as raw figures in Table II and as percentages of the entire consultation in Figures 2 and 3.

The consultations were on average 28 (± 10) min in length. Approximately one-third of the consultation was taken up with the doctor initiating speech, one-third with physical examination and one-third with interaction (questions and answers) between doctor and patient. Patients spoke for an average of 24% of the consultation and the doctor for 44%. The doctor made about 20 statements per consultation and answered approximately seven questions. Patients asked on average 5.6 (± 4.5) questions. Patient questions took up an average of 0.07% of the time the patient spent talking, i.e. the patient talked for 6.7 min but asked questions for only 32 s. The mean number of patient questions from those who did ask questions (6.23) and the mean number of oncologist responses to questions (6.6) were very similar (corr = 0.88), suggesting that patients received answers to their questions. Variability in both doctor and patient behaviour was quite high (see Table II).

Figure 3 shows a breakdown of the time during which the doctor or patient spoke. Patients talking about history and symptoms took up the longest time period (26.8% of speech time), followed by the doctor talking about treatment (25.5% of speech time). In five consultations (5%) there was no discussion of non-medical matters, while in 45 (49%) there were one or two speech units coded as social or 'other'. The median number of social units was 2.

Prognosis was discussed least (4.3% of speech time). In 33% of consultations, prognosis was not mentioned at all, while in a further 18.7% it was mentioned once. There were some identifying features of consultations in which prognosis was mentioned, although these were not statistically significant. Prognosis was more likely to be discussed if the patient had metastatic disease (74% *vs* 64% of those whose cancer was localised), if the patient was female (68% *vs* 58% of males) and if the patient had lung cancer (83% *vs* 73% of those with breast cancer and 59% of those with cervical cancer).

**Table II** Average duration and variability of doctor and patient behaviour in the cancer consultation

| | *Doctor behaviour* mean[a] (*s.d.*) | *range* | *Patient behaviour* mean (*s.d.*) | *range* |
|---|---|---|---|---|
| *Type of verbalisation* | | | | |
| Initiated statement | 8.7 (4.4) | 1.8–23.9 | 2.0 (1.5) | 0.0–6.8 |
| Question | 2.0 (1.1) | 0.1–4.8 | 0.5 (0.6) | 0.0–2.4 |
| Responses to question | 1.5 (1.6) | 0.0–8.4 | 4.2 (1.9) | 0.4–12.6 |
| *Content of verbalisation* | | | | |
| History/symptoms | 2.5 (0.9) | 0–4.6 | 5.1 (2.6) | 0.5–14.5 |
| Treatment | 4.8 (3.8) | 0–15.9 | 0.6 (0.7) | 0.0–3.8 |
| Diagnosis | 2.7 (1.7) | 0.2–7.6 | 0.3 (0.3) | 0.0–1.2 |
| Prognosis | 0.7 (0.9) | 0.0–4.6 | 0.1 (0.2) | 0.0–0.9 |
| Other | 1.4 (1.3) | 0.1–9.4 | 0.7 (2.6) | 0.0–3.1 |

[a] Mean duration expressed in minutes.

**Figure 2** Duration of consultation process.

**Figure 3** Duration of consultation content areas.

The ratio of doctor to patient talk ranged from 0.46 to 13.4, with a mean of 2.3 and a median of 1.8. The ratio was derived by dividing the duration of doctor talk by the duration of patient talk. Thus, a ratio of 2.3 indicates that the doctor talked for twice as long as the patient. Fifteen patients spoke for a longer period than the doctor; these patients were not noticeably different from the total sample on any of the demographic or disease variables.

Consultation style was scored on a linear analogue scale, on which the coder made a mark on a 100 mm line to indicate whether the doctor's behaviour was closer to the authoritarian (0 mm) or affiliative (100 mm) end. Overall, the consultation style was rated as being affiliative (mean = 82 mm). Similarly, the doctor was generally rated as friendly (mean = 85 mm) and relaxed (mean = 88 mm). Only one consultation was rated as more authoritarian than affiliative (LASA = 45 mm) and five (5%) were rated at 70 mm; these consultations involved a range of patients with no outstanding features. In no consultations was the doctor rated as less than 75 mm in relaxation and friendliness.

### *Behaviour of the doctor*

Age, sex, anxiety, involvement preference, in/out-patient status, type and stage of cancer and presence of family at the consultation were modelled as predictors of overall consultation style, doctor affect and interaction variables, using regression analysis and analysis of variance where appropriate. These variables were of experimental interest, or had been found in previous studies to relate to similar outcomes

(Cassileth, et al., 1980; Dunn et al., 1993). Time since diagnosis, occupation and marital status were not modelled, as univariate analyses showed no relationship between these variables and the outcomes. There was insufficient variability in *information* preference (only eight patients wanted less than all news), to model this variable meaningfully.

Overall, age, patient status and gender were the variables which most influenced the doctor and patient behaviour.

*Consultation style and doctor affect* The doctor was equally affiliative, relaxed and friendly with out-patients and in-patients, old and young, people from all occupations and with all types and stages of disease. Univariate analysis showed the doctor to be more affiliative with anxious patients ($t_{90} = 2.6$, $P < 0.01$) and female patients ($t_{89} = -2.4$, $P < 0.05$), but in multiple regression doctor affiliation was significantly related only to gender ($\beta = -4.6$, $P < 0.05$; $r^2 = 0.11$) (see Table III). The doctor was more affiliative with females than with males. In both univariate and multivariate analyses, only presence of a family member was significantly related to doctor relaxation ($\beta = 2.7$, $P < 0.05$, $r^2 = 0.09$). The doctor was more relaxed when a family member was not present. The doctor was rated as being equally friendly with all patients. As scores on consultation style and doctor affect were negatively skewed, we repeated the analysis with transformed categorical variables (median split). Gender remained significantly associated with affiliative behaviour ($\chi^2 = 8.9$, $P < 0.01$), while a trend to an association between presence of a family member and doctor relaxation was observed ($\chi^2 = 2.9$, $P < 0.1$).

*Length of time doctor spoke* Age interacted with patient status in determining how long the doctor talked during the consultation ($\beta = 3.8$, $r^2 = 0.23$, $P < 0.01$) (see Table III). For younger patients, patient status had little impact on the time the doctor spoke (14.3 min for out-patients *vs* 13.5 for in-patients). In contrast, in the older age group, patient status had a significant impact (12.5 min for out-patients *vs* 8.9 min for in-patients). The doctor spoke for a longer time to younger patients, regardless of patient status. It was not the number of speech items which differed according to age, but the time spent discussing each point ($t_{90} = -2.9$, $P < 0.01$). The doctor covered the same number of points with old and young patients, (on average 56) but spent longer discussing each point if the patient was young (13.1 s *vs* 9.9 s for older patients).

When the content of the consultation was analysed by individual categories, a number of differences emerged. The doctor spoke at greater length about history and symptoms, diagnosis and non-medical matters to out-patients than to in-patients ($\beta = -51.9$, $P < 0.0001$; $\beta = -59.5$, $P < 0.01$; $\beta = -34.5$, $P < 0.05$ respectively), but spoke for an equal length of time about prognosis and treatment. He spoke at greater length about prognosis and treatment ($P < 0.01$) and answered more questions ($P < 0.05$) with younger patients than with those who were older.

In univariate analysis, the doctor also spoke for a significantly longer time to patients who wanted involvement in decision-making (Student $t_{77} = -2.2$, $P < 0.05$), and when a family member was present (Student $t_{58} = 2.5$, $P < 0.05$), although these effects were no longer significant in multiple regression.

### *Behaviour of the patient*

Patients wanting involvement in medical decision-making spent more time asking questions ($P < 0.001$), and initiating statements ($P < 0.001$). Age ($P = 0.01$), in/out-patient status ($P < 0.01$) and gender ($P < 0.05$) were predictive of both number and duration of patient questions, explaining 15% of the variance for both outcomes. Younger people, females and out-patients asked more questions and spent longer doing so. Table IV shows the mean number of questions within the six content areas by age, sex and out/in-patient status. Questions about diagnosis and treatment primarily differentiated between groups, although out-patients also asked more questions about social matters (in-patients never discussed social matters).

**Table IV** Mean number of questions asked within seven content categories

| *Question Categories* | *Old*[a] | *Young* | *Male* | *Female* | *In-patient* | *Out-patient* |
|---|---|---|---|---|---|---|
| Treatment | 2.4** | 3.9 | 1.6 | 3.3 | 2.8 | 3.2 |
| Diagnosis | 0.8* | 1.6 | 0.4* | 1.3 | 0.6** | 1.6 |
| Prognosis | 0.2 | 0.3 | 0.2 | 0.3 | 0.2 | 0.2 |
| History/presenting symptoms | 0.2 | 0.4 | 0.2 | 0.3 | 0.2 | 0.4 |
| Other medical matters | 0.5 | 0.7 | 0.4 | 0.6 | 0.4 | 0.8 |
| Social matters | 0.2 | 0.1 | 0.1 | 0.2 | 0.0* | 0.3 |

[a]Median split at age 53. *$P<0.05$; **$P<0.01$.

**Table III** Variations in consultation style, doctor affect and duration of doctor speech as a function of patient characteristics

| *Patient characteristic* | n | *Authoritarian* vs *affiliative Mean (s.d.)*[1] | *Anxious* vs *relaxed Mean (s.d.)*[a] | *Hostile* vs *friendly Mean (s.d.)*[a] | *Duration of doctor talk Min (s.d.)* |
|---|---|---|---|---|---|
| Whole sample | 142 | 82 (7.0) | 85 (4.8) | 88 (3.8) | 12.3 (5.4) |
| Sex | | | | | |
| Male | 18 | 78 (5.5) | 82 (4.6) | 86 (4.8) | 12.4 (5.0) |
| Female | 124 | 83 (12.7)** | 85 (5.4) | 89 (3.6) | 12.3 (5.4) |
| Family | | | | | |
| Present | 36 | 81 (5.7) | 84 (4.6) | 87 (3.7) | 13.9 (5.5)* |
| Not present | 106 | 83 (5.2) | 86 (4.0) | 89 (3.1) | 9.5 (4.0) |
| Involvement preference | | | | | |
| Yes | 93 | 81 (7.5) | 84 (4.6) | 87 (3.7) | 13.9 (5.5)* |
| No | 33 | 84 (4.9) | 86 (4.0) | 89 (3.1) | 9.5 (4.0) |
| Age[b] | | | | | |
| Old | 75 | 82 (5.8) | 85 (4.7) | 88 (4.4) | 10.7 (4.9) |
| Young | 67 | 82 (8.4) | 85 (4.9) | 88 (3.2) | 14.3 (5.2)** |
| Anxiety | | | | | |
| High | 72 | 84 (5.7) | 84 (4.9) | 88 (3.7) | 13.5 (5.5) |
| Low | 70 | 80 (7.9)** | 85 (4.7) | 88 (3.8) | 10.9 (4.9) |

[a]Scores are based on 100 mm LASA lines with the first descriptor at the left (0 mm) and the second at the right end of the line (100 mm) [b]Median split. * $P<0.05$, ** $P<0.01$.

*Consultation outcomes*

Psychological adjustment, satisfaction and recall were not related to the total length of the consultation. Nor were these outcomes affected by consultation style, doctor affect, the duration of conversation about non-medical matters or the ratio of doctor to patient talk.

Patients whose questions were answered (i.e. who had consultations in which the ratio of patient questions to doctor responses to questions was lower) felt that cancer had less of an impact on their daily lives ($t_{62} = -2.5$, $P < 0.01$). Satisfaction and recall were not affected by the ratio of patient questions to doctor responses to questions.

### Discussion

The CN-LOGIT computerised interaction analysis system was used in this study to examine the consultations of a single oncologist. It was possible to describe the consultations in great detail, in terms of the frequency and duration of content areas and forms of language. Intra-rater reliability was excellent and inter-rater was satisfactory. We are currently revising our manual and training procedures to ensure an improvement in the latter.

Although doctors report that they vary their consulting style depending on the patient, the data of Roter *et al.* (1987) indicated that doctors behaved consistently with different patients. We were able to demonstrate significant differences in consultation style (affiliative *vs* authoritarian, as rated by an independent coder), doctor affect and duration of doctor talk as a function of patient characteristics. The doctor was also more affiliative with patients who reported high anxiety on the Spielberger State Anxiety Scale. The doctor was not privy to patient scores at the time of the consultation, and so presumably reacted to behavioural cues in differentiating between patients on these bases.

These are variations in consultation behaviour which have face validity, suggesting not only that the doctor concerned was communicating sensitively with his patients, but also that the CN-LOGIT interaction analysis was able to highlight some important aspects of the consultation.

Patients who expressed a desire for involvement in medical decision-making spent more time asking questions and making statements. Female patients, younger patients and outpatients also asked more questions. The fact that the doctor spent more time responding to questions and talking overall to these patients suggests an interactive process. CN-LOGIT retains the sequence of events, as well as how much of a particular ingredient is present, and in future analyses we will explore sequential information effects.

Much of the communication literature emphasises the importance of the patient-centred consultation style, in which the patient's point of view is actively sought by the physician and allowed to direct the proceedings. We measured this concept in a number of ways; the ratio of doctor to patient talk, the ratio of patient questions to doctor responses to questions, discussion of social and non-medical matters and the subjective rating of consultation style. Conversation about non-medical matters, length of the consultation, rated consultation style and patient activity in the consultation did not influence patient satisfaction, recall or psychological adjustment. This is surprising given positive findings elsewhere (Stewart, 1984; Roter *et al.*, 1987; Maguire and Faulkner, 1988). It is possible that the consultation style of the single oncologist involved in this study was not significantly variable along these dimensions. We are planning to repeat this study with 12 medical and radiation oncologists to explore a variety of doctors, patients, situations and consultation styles.

Our data on one indicator provided support for the notion that patient-centred consultations will lead to improved patient outcomes. Patients whose questions were answered showed better psychological adjustment 3 weeks after the consultation than those who asked questions but did not receive a response. These data support a number of studies of general practice consultations, which found that patient-centred skills, such as answering questions, counselling and encouraging patients to talk, were positively related to satisfaction (Stewart, 1984; Roter *et al.*, 1987; Bertakis *et al.*, 1991).

In summary, this study provides evidence of the validity and power of the CN-LOGIT computerised interaction system. Close examination of the cancer consultation via this technique offers the potential to provide specific recommendations for effective communication with cancer patients. With increased emphases in medical curricula on doctor–patient communication (Maguire *et al.*, 1986) and the desire to minimise medicolegal complaints, these data are essential for an evidence-based approach to communication in the cancer consultation and, indeed, to a wide variety of medical situations.

Revision of the system is under way to take into account evidence of the importance of more subtle interaction factors. For example, Maguire (1985) has reported that the level of emotional disclosure by the patient is an important marker of the level of skill of the doctor in eliciting concerns. Further research will explore whether the current findings supporting the patient-centred consultation can be applied to the wider oncology setting.

**Acknowledgments**

This work was undertaken with financial assistance from the NSW State Cancer Council. We thank Ms Roslyn Sparrevohn for her assistance in coding the data.

### References

ANDERSON SO AND MATTSSON B. (1989). Length of consultation in general practice in Sweden: views of doctors and patients. *Fam. Pract.*, **6**, 130–135.

BAIN DJG. (1977). Patient knowledge and the content of the consultation in general practice. *Med. Educ.*, **11**, 347.

BALES RF. (1950). *Interaction Process Analysis*. Addison Wesley: Cambridge.

BERTAKIS KD, ROTER D AND PUTNAM SM. The relationship of physician medical interview style to patient satisfaction (1991). *J. Fam. Pract.* **2**, 175–181.

BLANCHARD CG, LABRECQUE MS, RUCKESCHEL JC AND BLANCHARD EB. (1990). Physician behaviours, patient perceptions and patient characteristics as predictors of satisfaction of hospitalized adult cancer patients. *Cancer*, **65**, 186–192.

BLUM RH. (1960). *The Management of the Docot-patient Relationship*. McGraw-Hill; New York.

BUTOW P, COATES A, DUNN S, BERNHARD J AND HURNEY C. (1991). On the receiving end IV. validation of quality of life indicators. *Ann. Oncol.*, **2**, 597–603.

BUTOW PN, DUNN SM, TATTERSALL MHN AND JONES Q. (1994). Patient participation in the cancer consultation: evaluation of a question prompt sheet. *Ann. Oncol.*, **5**, 199–204.

CARTWRIGHT A. (1964). *Human Relations and Hospital Care*. Routledge & Kegen Paul: London.

CASSILETH BR, ZUPKIS RV, SUTTON-SMITH K AND MARCH V. (1980). Information and participation preferences among cancer patients. *Ann. Intern. Med.*, **92**, 832–836.

CHIATCHIK S, KREITLER S, SHAKED S, SCHWARTZ I AND ROSIN R. (1992). Doctor–patient communication in a cancer ward. *J. Cancer Educ.*, **7**, 41–54.

COHEN F, LAZARUS RS. (1979). Coping with the stresses of illness. In Health Psychology, Stone GC, Cohen F, Adler NE. (eds). pp. 217–254. Jossey-Bass: San Francisco.

DEROGATIS LR, MORROW GR, FETTING J, PENMAN D, PIASETSKY S, SCHMALE AM, HENRICHS M AND CARNICKE CL. (1983). The prevalence of psychiatric disorders among cancer patients. *JAMA*, **23249**, 751–757.

DUNN SM, PATTERSON PU, BUTOW PN, SMARTT HH, McCARTHY WH AND TATTERSALL MHN. (1993). Cancer by another name: a randomised trial of the effects of euphemism and uncertainty in communicating with cancer patients. *J. Clin. Oncol.*, **11(5)**, 989-996.

DUNN SM, BUTOW PN, TATTERSALL MHN, JONES QJ, SHELDON JS, TAYLOR JJ AND SUMICH MD. (1993). General information tapes inhibit recall of the cancer consultation. *J. Clin. Oncol.*, **11**, 2279-2285.

HOLSTI OR. (1969) *Content Analysis for the Social Sciences and Humanities*. Aldison-Wesley: Reading, MA.

INUI TS, CARTER WB, KUKULL WA AND HAIGH VH. (1982). Outcome-based doctor-patient interaction analysis. *Med. Care*, **20**, 535-549.

KIDDLER LH. (1986). *Research Methods in Social Relations*. Holt, Rinehart & Winston: New York.

KORSCH BM, GOZZI EK AND FRANCIS V. (1968). Gaps in doctor-patient communication. *Pediatrics*, **42**, 855,1968.

LEY P. (1988). *Communicating with Patients; Improving Communication, Satisfaction and Compliance*. Croom Helm; New York.

LEY P AND SPELMAN MS. (1965). Communications in an outpatient setting. *Br. J. of Soc. Clin. Psych.*, **4**, 114-116.

LAY P, BRADSHAW PW, EAVES D AND WALKER CM. (1973). A method for increasing patients' recall of information presented by doctors. *Psychol. Med.*, **3**, 217-220.

LINN LS AND GREENFIELD S. (1981). Patient suffering and patient satisfaction among the chronically ill. *Med. Care*, **20**, 425-431.

McNAIR DM. (1981). *EITS Manual for the Profile of Mood States*. EITS: San Diego.

MAGUIRE P. (1985). Barriers to psychological care of the dying. *Br. Med. J. Clin. Res. Educ.*, **291**, 1711-1713.

MAGUIRE P AND FAULKNER A. (1988). How to do it. Communicate with cancer patients: 1. Handling bad news and difficult questions. *Br. Med. J.*, **297**, 907-909.

MAGUIRE P, FAIRBURN S AND FLETCHER C. (1986). Consultation skills of young doctors: I. Benefits of feedback training in interviewing as students persist. *Br. Med. J. Clin. Res. Educ.*, **392**: 1573-1576.

MORROW GR, HOAGLAND AC AND CARPENTER PJ. (1983). Improving physician-patient communications in cancer treatment. *J. Psychosoc. Oncol.*, **1(2)**, 93-101.

NSW Department of Health, Complaints Unit. (1991, 1992, 1993). *Annual Reports*. Department of Health: Sydney.

ROTER DL. (1977). Patient participation in patient-provider interactions: the effects of patient question-asking on the quality of interactions, satisfaction and compliance. *Health Educ. Monogr.* **5**, 281-312.

ROTER DL. (1984). Patient question asking in physician-patient interaction. *Health Psychol.* **3**, 395-409.

ROTER DL, HALL JA AND KATZ NR. (1987). Relations between physician behaviours and analogue patients' satisfaction, recall and impressions. *Med. Care*, **25**, 437-451.

SCHIPPER H, CLINCH J, McMURRAY A AND LEVITT M. (1984). Measuring the quality of life of cancer patients: the functional living index - cancer: development and validation. *J. Clin. Oncol.*, **2**, 472-483.

SPIELBERGER CD. (1983). Manual for the state-trait anxiety inventory STAI (Form Y). Consulting Psychologists Press: Palo Alto, CA.

STEWART M. (1984). What is a successful doctor-patient interview? A study of interactions and outcomes. *Soc. Sci. Med.*, **19**, 167-175.

SUTHERLAND HJ, LLEWELLYN-THOMAS HA, LOCKWOOD GA, TRITCHLER DL AND TILL JE. (1989). Cancer patients: their desire for information and participation in treatment decisions. *J. R. Soc. Med.*, **82**, 260-3.

TATTERSALL MHN, BUTOW PN, GRIFFIN A-M AND DUNN SM. (1994). The take-home message after a cancer consultation: a randomised trial of consultation audiotapes and individualised letters to patients. *J. Clin. Oncol.*, **12**, 1305-1311.

WASSERMAN RC AND INUI TS. (1983). Systematic analysis of clinical-patient interactions: A critique of recent approaches with suggestions for future research. *Med. Care*, **11**, 279-293.

ZIGMOND AS AND SNAITH RP. (1983). *The Hospital Anxiety and Depression Scale*. *Acta Psychiatr. Scand.*, **67**, 362-370.